# The Sympatric Coexistence Mechanism: A Case Study of Two Penahia Species in the Beibu Gulf, South China Sea

**DOI:** 10.3390/ani14060849

**Published:** 2024-03-09

**Authors:** Konglan Luo, Xiaodong Yang, Yan Zhou, Xiaoying Yi, Chunxu Zhao, Jinxi Wang, Xiongbo He, Yunrong Yan

**Affiliations:** 1 Fisheries College, Guangdong Ocean University, Zhanjiang 524088, China; luokonlan@gmail.com (K.L.); yangxd2832@163.com (X.Y.); 13610513561@163.com (Y.Z.); xiaoyingzis2024@163.com (X.Y.); jinxiwang96@126.com (J.W.); 2 Southern Marine Science and Engineering Guangdong Laboratory (Zhanjiang), Zhanjiang 524057, China; ct9zcx@163.com; 3 Guangdong Provincial Engineering and Technology Research Center of Far Sea Fisheries Management and Fishing of South China Sea, Guangdong Ocean University, Zhanjiang 524088, China

**Keywords:** *Pennahia pawak*, *Pennahia anea*, stomach content analysis, stable isotope analysis, sympatric coexistence

## Abstract

**Simple Summary:**

In this study, the feeding habits, trophic niches, and spatial niches of *Pennahia pawak* and *Pennahia anea* were investigated with the aim of exploring the coexistence and competition mechanism of these two species in the Beibu Gulf. The results indicate that both *P. pawak* and *P. anea* exhibit feeding shifts and differentiation in their trophic and spatial niches.

**Abstract:**

The study of trophic relationships among closely related species plays an important role in deepening our understanding of the resource utilization characteristics, differentiation patterns, and population dynamics of co-occurring species in the same habitat. This research uses two congeneric fish species, *Pennahia pawak* and *Pennahia anea*, as examples. Based on a stomach content analysis and a carbon–nitrogen stable isotope analysis, a comparative analysis of their feeding habits and trophic niches is conducted. Additionally, a spatial niche analysis is employed to explore the coexistence and competitive mechanisms between these two closely related fish species. The results show that specialized feeding habits mitigate intraspecific competition as the population densities increase. The carbon and nitrogen stable isotope analysis reveals variations in the feeding habits and trophic levels with body length, indicating adaptive shifts in prey selection. Despite similar food resources, niche differentiation arises due to differences in dominant prey, facilitating coexistence. Differences in spatial niche further contribute to niche separation and coexistence. In resource-limited environments, species such as *Pennahia* utilize trophic and spatial niche differentiation to collectively exploit resources and achieve coexistence, with implications for fishery management favoring *Pennahia* resource occupancy capabilities.

## 1. Introduction

Competition is defined as a rivalry where two or more parties strive for limited resources. Competition for food and space between different species is relatively common in communities, and the result is almost always a victory for one side and the exclusion of the other. Two species with similar food habits compete for any limited resource, but it is common for species to compete for food resources [1,2]. Food competition is one of the important manifestations of food relationships, including intraspecific and interspecific food competition. Intraspecific food competition can be broadly defined as the effort made by two or more individuals of the same species to access a crucial and limited food resource. In the short term, competing individuals may adopt different behavioral strategies and feeding patterns or establish dominance hierarchies and feeding areas; in the long term, intraspecific food competition can drive ecological role turnover [3]. Studying the similarity in the compositions of prey organisms consumed by different organisms (overlapping feeding niches) can help us understand food competition between species; a higher the degree of overlap in food between fish species leads to competition for food; on the contrary, if there is little food overlap between fish species, this means that the greater the differences in prey, the less competition between species. When prey organisms in the environment are limited, fish with similar niches will have severe food competition, which may lead to the turnover of dominant species and changes in the community structure [4]. The study of interspecific food relationships among fishes of the same genus in the same sea area can reflect the status of fish utilization of food resources and the existence of competition in the feeding process, and this is the basis for studying the trophic dynamics of food webs [5]. Many scholars, both domestically and internationally, have conducted research on fish feeding relationships [6,7,8,9,10], but most of them have focused on relationships between different species or non-congeneric species. The interrelationships among closely related species are particularly unique and significant aspects of species relationships [11]. Due to the advancements in theoretical research and scientific technology, there is an increasing focus on the study of food relationships among closely related species [12,13,14]. It is anticipated that this attention will lead to a deeper understanding of the mechanisms underlying species coexistence.

Feeding relationship research tools mainly include the traditional stomach content analysis and stable isotope analysis [15,16]. A stomach content analysis is a traditional method that has been used for a long time in marine feeding ecology, and its advantage is that it can accurately and intuitively reflect the composition and taxonomic characteristics of the prey consumed by fish [17]. However, a traditional stomach content analysis is time-consuming and labor-intensive, and it has the drawbacks of only being able to characterize transient feeding information as well as not being able to determine the fragmented prey organisms being digested, whereas stable isotopes of carbon and nitrogen in tissues, which can record feeding information on medium to long time scales, have been proven to be the best alternatives and complements to stomach content analysis [18], and they have been widely used in the reconstruction of feeding habits of aquatic organisms. However, the stable isotope technique also has some limitations in that it is unable to visually obtain information on ingested food types and sizes, so a stable isotope analysis and stomach content analysis were combined as complementary techniques to more comprehensively and accurately reflect the food relationships of organisms in the study of feeding ecology.

*Pennahia pawak* and *Pennahia anea* have relatively important ecological niches as relatively stable resource fish species in the Beibu Gulf [19]. Studies on *P. pawak* and *P. anea* have mainly focused on fishery biology [20,21], growth and mortality [22], reproductive biology [23], population structural characteristics and spatial–temporal distribution [24,25], and feeding ecology [26], whereas studies on the interspecies feeding relationship between *P. pawak* and *P. anea* have not been reported. These two co-distributed species are not only closely related, but also similar in size, shape, and ecology [19]. So, there may be potential feeding competition between them. In this study, using a stomach content analysis and a carbon–nitrogen stable isotope analysis, we compared and analyzed the differences in the feeding habits and trophic niches of *P. pawak* and *P. anea*. We included a spatial niche analysis to elucidate the feeding relationship between these two closely related species, aiming to explore the mechanisms of coexistence of sympatric species in the Beibu Gulf.

## 2. Materials and Methods

### 2.1. Sample Collection and Processing

**Figure 1:**
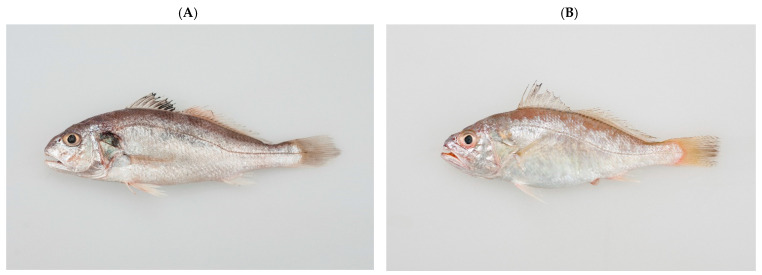
Images of *Pennahia pawak* (**A**) and *Pennahia anea* (**B**) in Beibu Gulf, South China Sea.

Samples for trophic niche analysis were collected in 2020 from Guangdong Jianghong and Beihai fishing harbors, with 1506 and 1684 samples of *P. pawak* and *P. anea*, respectively, which were randomly sampled in four seasons (Table 1). The samples collected in each season were frozen and sent back to the laboratory for the next step of processing. Further classification and identification of the collected samples will be conducted to determine the correct research species (Figure 1). After thawing the samples in the laboratory, biological indicators such as total length, body length, and weight were measured according to Specifications for oceanographic survey [27], and stomach content samples were retained for freezing and preservation.

### 2.2. Stomach Contents Analysis

After thawing the stomach content samples, the food mass was removed and placed in a clean Petri dish, and the excess water on the surface of the food mass was carefully blotted out with filter paper. Subsequently, efforts were continued to identify the various species of prey within the food mass, aiming to classify these prey organisms down to the finest taxonomic order possible. The food mass was weighed (to the nearest 0.01 g), and the data were recorded.

In this study, the repletion index (*RI*) and vacuity coefficient (*VC*) were used to determine the feeding intensity of fish [28]. The formulas are as follows:

(1) $$\mathrm{RI}=\frac{\sum W_{\mathrm{if}}}{W_{f}}$$

(2) $$\begin{array}{c} VC=\frac{\mathrm{no}.\;\mathrm{of}\;\mathrm{empty}\;\mathrm{stomachs}}{\mathrm{total}\;\mathrm{no}.\;\mathrm{of}\;\mathrm{stomachs}} \end{array}$$

where *W_f_* is the total weight, *f*, of the fish, and $\sum W_{if}$ is the total weight, *f*, of all the food in the stomach of the fish and the vacuity coefficient (*VC*).

The importance of prey organisms was evaluated using the Index of Relative Importance percentage (*IRI%*) [29] with the following formula:

(3) $$\begin{array}{c} IRI=\left(N+W\right)\times F \end{array}$$

(4) $$\begin{array}{c} IRI\%=\left(\frac{IRI}{\sum IRI}\right)\times 100\% \end{array}$$

where *N* is the number of a given prey as a percentage of the total number of prey; *W* is the weight of a given prey as a percentage of the total weight of prey; and *F* is the percentage of frequency of occurrence of each prey.

The Shannon–Wiener diversity index (*H*′) was used to evaluate the width of the trophic niche of the two fish species. The overlap of trophic niches between the two species [30,31] was also calculated by using Pianka’s coefficient of ecosystem overlap (*O_ij_*) to evaluate the intensity of competition between the two species.

(5) $$\begin{array}{c} H_{i}^{\prime }=-\Sigma P_{ik}ln\left(P_{ik}\right) \end{array}$$

$H_{i}^{\prime }$ is the width of the trophic niche for fish *i*, and *P_ik_* is the percentage of the number of prey *k* in the food of fish *i*(N).

(6) $$\begin{array}{c} O_{ij}=\frac{\Sigma_{k=1}^{s}P_{ik}\times P_{jk}}{\sqrt{{\Sigma_{k=1\;}^{s}P}_{ik}^{2}\times \Sigma_{k=1}^{s}P_{jk}^{2}}} \end{array}$$

where *s* is the total number of prey species consumed by the two fish species, and *P_ik_* and *P_jk_* represent the number percentage (N) of prey *k* in the prey composition of fishes *i* and *j*, respectively. The value of *O_ij_* ranges from 0 to 1. The larger the value, the higher the food similarity, that is, the fiercer the food competition. *O_ij_* > 0.3 means that the overlap is effective, and *O_ij_* > 0.6 has significant overlap.

Individual specialization (IS) was used to evaluate the intensity of intraspecific competition between the two species [32], and the Whin-individual component (WIC) to the width of its trophic niche (*H*′) was calculated. The range was 0–1, and the smaller the ratio, the lower the individual overlap and the higher individual specialization [33]. The related calculation formula is as follows:

(7) $$\begin{array}{c} WIC=\sum_{i}P_{i}(-\sum_{k}P_{ik}ln(P_{ik})) \end{array}$$

(8) $$\begin{array}{c} IS=WIC/H_{i}^{\prime } \end{array}$$

where *P_i_* is the percentage of prey consumed by an individual *i* to the population of prey in that population, and *P_ik_* is the percentage of prey consumed by an individual *i* to the population of prey in that population.

### 2.3. Carbon and Nitrogen Stable Isotope Analysis

A rectangular cut was made below the dorsal fin and above the lateral line of the fish, the fish epidermis was peeled off from the inner side of the skin, and an appropriate amount of muscle sample was taken from the notch in a 2 mL centrifuge tube and cryopreserved for subsequent processing.

The muscle sample was placed in the sample tray of the freeze dryer (Christ, Osterode, Germany, Alpha1-4/2-4LD Plus), and it was freeze-dried for 48 h at −48 °C until the muscle maintained a constant weight. The sample was taken out, and 2 small steel balls were put into each centrifuge tube containing the sample. Then, it was put in the homogenizer (BIOSPEC MiniBeadbeater-16, Biospec, Bangor, PA, USA) and ground for 1 min. The ground muscle powder was embedded and sent to the Isotope Laboratory of the School of Marine Meteorology, Guangdong Ocean University, where it was measured using an EA Isolink Elemental Analyzer (Thermo Fisher scientific, Waltham, MA, USA) and a 253 Plus Isotope Mass Spectrometer (Thermo Fisher scientific, Waltham, MA, USA).

The stable isotope ratios are expressed according to the internationally accepted δ-value [34], which is calculated using the following formula:

(9) $$\begin{array}{c} \delta X=(\frac{R_{\mathrm{sample}}}{R_{\mathrm{standard}}}\;-1)\times 1000 \end{array}$$

where *δX* denotes the carbon stable isotope ratio δ^13^C or nitrogen stable isotope ratio δ^15^N, *R_sample_* denotes the ratio of carbon or nitrogen stable isotope in the sample, and *R_standard_* denotes the ratio of carbon or nitrogen stable isotope in the standard. The carbon isotope ratio is ^13^C/^12^C, and the nitrogen stable isotope ratio is ^15^N/^14^N.

The trophic level formula is as follows [35]:

(10) $$\begin{array}{c} TL=\frac{\delta^{15}N_{\mathrm{sample}}-\delta^{15}N_{0}}{\delta^{15}N_{c}}+{\mathrm{TL}}_{b} \end{array}$$

where *TL* denotes the trophic level of the fish; *δ^15^N_sample_* denotes the nitrogen stable isotope signature of the sample; *δ^15^N_0_* is the nitrogen stable isotope signature of the baseline organism; *δ^15^N_c_* denotes the nitrogen isotope enrichment (3.4‰); and *TL_b_* denotes the trophic level of the baseline organism. According to our team’s pre-monitoring study, *Amusium pleuronectes* are qualified as baseline organisms because they have stable feeding habits, longevity, and ease of sampling, feeding mainly on plankton and organic detritus [36]. Therefore, we chose them as the baseline organisms for calculating the trophic level in this study. Their nitrogen isotope value was determined to be 8.89‰, and the trophic level was set at 2.

The degree of overlap between SEAc was used to quantify the area of trophic niche overlap between *P. pawak* and *P. anea* [35], and the overlap of the two species was expressed as the proportion of overlap in the maximum likelihood estimate of SEAc, which represents the point estimate of trophic niche overlap between the two species, with an overlap close to 0 indicating that the two ellipsoids are separated, and a value close to 1 indicating that the two ellipsoids are completely overlapped. According to Schoener’s D index and according to the results of other stable isotope niche area overlap studies [37], *B_ij_* is the niche overlap index, ranging from 0 to 1, with larger values indicating higher overlap, and a *B_ij_* value greater than 0.3 is regarded as a meaningful overlap, and a value greater than 0.6 is regarded as a significant overlap [38].

### 2.4. Spatial Niche

The summer close season is from 12:00 a.m. May 1 to 12:00 a.m. August 16. The data used for the spatial niche analysis came from surveys of 26 stations in the Beibu Gulf before (April) and after (August) the period of fishing moratorium. The survey vessel had a power of 441 KW, and the net was a bottom trawl with a 20 m wide net opening, a maximum mesh of 5 cm, and a sac mesh of 2 cm. One hour of trawling was conducted at each station. Samples were collected and preserved on ice and brought back to the laboratory for classification and biological determination. The number and weight of each species were recorded and converted to biomass (kg/km^2^) and abundance (ind/km^2^) per unit area. Sample sampling and analysis were performed according to Specifications for oceanographic survey [27].

(1) Resource intensity [39]

(11) $$\begin{array}{c} D=\frac{C}{a\left(1\;-\;E\right)} \end{array}$$

where *D* is the resource density (kg·km^−2^); *C* is the catch rate (kg·h^−1^); *E* is the escape rate taking the empirical value (0.5) [24]; and *a* is the hourly swept area (km^2^) of the survey vessel, where the swept width is taken as 1/2 of the length of the float line, and the towing speed is taken as the average towing speed of 3.0 kn.

(2) Spatial niche width and overlap

Spatial niche width and overlap were estimated based on catch, where spatial niche width was expressed using the Shannon–Wiener index (*H′*) [39], and the spatial niche overlap index (*Q_ij_*) was expressed using the Pianka index [30] with the following formula:

(12) $$\begin{array}{c} H^{\prime }=-\sum \left(P_{\mathrm{ij}}\ln P_{\mathrm{ij}}\right) \end{array}$$

(13) $$\begin{array}{c} Q_{\mathrm{ij}}=(\sum P_{\mathrm{ij}}P_{\mathrm{ik}})/\sqrt{\sum P_{\mathrm{ij}}^{2}\sum P_{\mathrm{ik}}^{2}} \end{array}$$

where *H′* denotes the spatial ecological niche width, and *P_ij_* denotes the mass percentage of fish *i* in the total biomass of the jth station. *Q_ij_* is the ecological niche overlap index, which ranges from 0 to 1, with larger values indicating a higher degree of overlap; specifically, a *Q_ij_* value greater than 0.3 is regarded as a meaningful overlap, and a value greater than 0.6 is regarded as a significant overlap [38].

### 2.5. Data Processing

Data were processed, analyzed, and graphed using Excel 2016 and SPSS 25.0, and Pearson analysis was used to test for linear correlation between body length and trophic level for two *Pennahia* species. Trophic niche of δ^13^C and δ^15^N of the two *Pennahia* species was determined using the SIAR [40] data package in R 3.6.3. And mean centrifugal distance (CD), carbon range (CR), nitrogen range (NR), mean nearest neighbor distance (MNND), standard deviation of nearest neighbor distance (SDNND), standard ellipse corrected area (SEAc), the area of the convex hull (TA), and other ecological indicators were also used todescribe the trophic niche.[35,38]. The distribution of resources was mapped using ArcGIS 10.8 software.

## 3. Results

### 3.1. Feeding Habit

#### 3.1.1. Differences in Feeding Habits within Species

**Figure 2:**
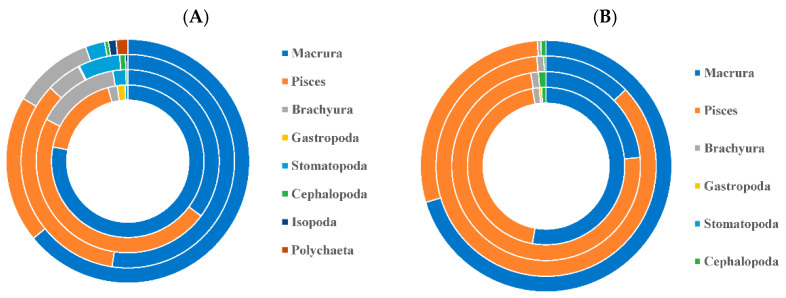
Diet composition by season for *Pennahia pawak* (**A**) and *Pennahia anea* (**B**) in Beibu Gulf. Circles are in order from inside out: spring, summer, fall, and winter. Eight kinds of prey are displayed with different colors, respectively.

**Figure 3:**
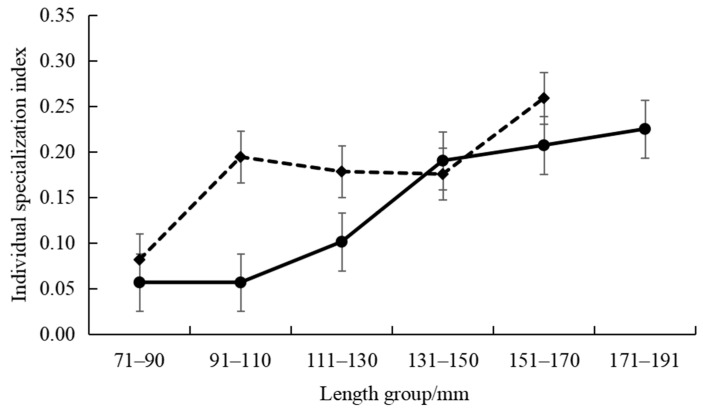
Individual specialization index variations of *Pennahia pawak* (solid line) and *Pennahia anea* (dashed line) in different length groups.

The intraspecific feeding habits of *P. pawak* varied significantly between seasons. In the spring and winter, the stomach contents of *P. pawak* had the highest proportions of Macrura (77.99% and 63.78%, respectively), and in the summer and autumn, they had the highest proportions of Pisces (47.37% and 34.73%, respectively); Gastropoda were also present only in the spring (Figure 2). The prey overlap coefficients among the length groups of *P. pawak* were all greater than 0.6 (except for 171–191 mm), and there was significant food overlap. The prey overlap coefficients between the 71–80 mm length group and the 91–110 mm length group, the 111–130 mm length group, and between the 91–110 mm length group and the 111–130 mm length group were close to 1, with an almost complete overlap of prey species (Table 2). Meanwhile, the individual specialization index of the groups of different lengths ranged from 0.06 to 0.22, with an overall increasing trend with the increase in length (Figure 3).

The intraspecific feeding habits of *P. anea* differed markedly between seasons. The highest proportions of Macrura in the stomach contents of *P. anea* were found in the spring and winter, and the highest proportions of Pisces were found in summer and autumn; in addition, gastropods were present only in the spring (Figure 2). The *P. anea* prey overlap coefficients were greater than 0.6 between the length groups, except between the 171–190 mm length group and the 111–130 mm length group, the 131–151 mm length group, and the 151–170 mm length group, which were less than 0.6, and there was significant food overlap (Table 2). Among them, the highest prey overlap coefficient was found between the 71–90 mm length group and the 171–190 mm length group, followed by the 131–150 mm length group and 151–170 mm length group. Meanwhile, the fluctuation range of the individual specialization index of different length groups was 0.08–0.26, which showed a fluctuating upward trend with the increase in length (Figure 3).

#### 3.1.2. Differences in Feeding Habits between Species

*P. pawak* and *P. anea* have similar feeding habits and some feeding preferences. The following are some of the most common species in the genus (Table 3). Although both species of *Pennahia* genus take Pisces and Macrura as prey, the proportion of Pisces in the prey of *P. anea* is obviously higher than that of *P. pawak*; there are also some differences in specific species of the two species of *Pennahia* genus, among which *P. anea* are dominated by *Alpheus*, *Bregmaceros*, and *Stolephorus*, whereas for *P. pawak*, in addition to *Alpheus* and *Bregmaceros*, *Metapenaeopsis barbata* and *Alpheus bisincisus* also accounted for certain proportions. The trophic niche widths for *P. pawak* and *P. anea* were 2.21 and 2.29, respectively, and the prey overlap coefficient was 0.56, reaching the level of meaningful overlap.

### 3.2. Isotopic Characteristics and Trophic Levels

**Figure 4:**
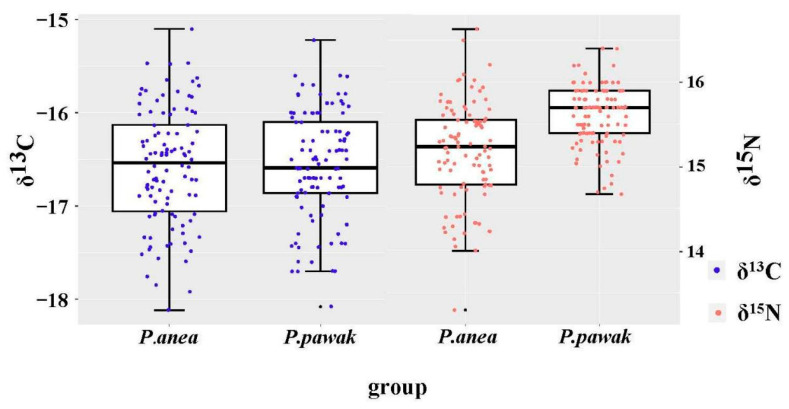
Stable isotopic distribution of δ^13^C (blue dots) and δ^15^N (pink dots) in *Pennahia pawak* and *P. anea*.

The δ^13^C and δ^15^N isotopic characteristics of *P. pawak* and *P. anea* are different to some extent. The δ^13^C of *P. pawak* ranged from −18.08 to −15.22‰, with a mean value of −16.57‰, and the δ^15^N ranged from 14.68 to 16.40‰, with a mean value of 15.60‰ (Figure 4). After the Pearson analysis, the length of *P. pawak* had a significant positive correlation with δ^15^N (Pearson r = 0.65, *p* < 0.01), but no significant correlation with δ^13^C (*p* > 0.05); the δ^13^C of *P. anea* ranged from −18.12 to −15.10‰, with a mean value of −16.58‰, and δ^15^N ranged from 13.65 to 16.63‰, with a mean value of 15.18‰ (Figure 4). After the Pearson analysis, the length of *P. anea* had a significant positive correlation with δ^15^N (Pearson r = 0.44, *p* < 0.01), while there was no significant correlation with δ^13^C (*p* > 0.05). The δ^13^C ranges of *P. pawak* and *P. anea* were similar, with that of *P. anea* being slightly higher, while *P. pawak* had a larger δ^15^N range than *P. anea*.

**Figure 5:**
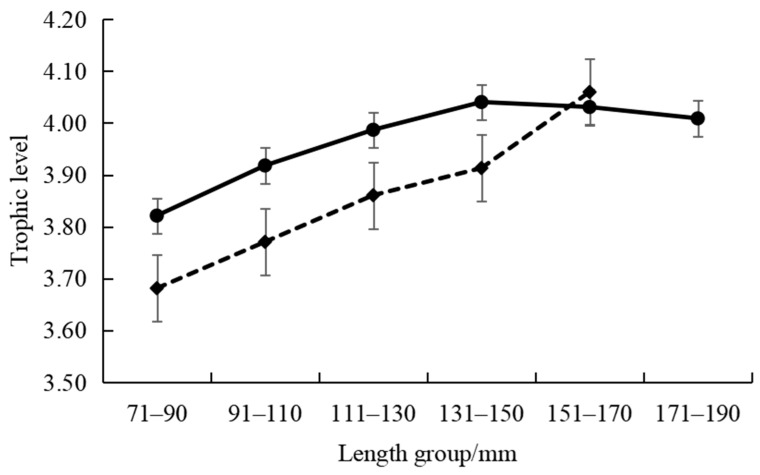
Change in trophic levels of *Pennahia pawak* (solid line) and *P. anea* (dashed line) in different lengths (length group, mm).

The one-way ANOVA showed that the trophic level differences between *P. pawak* and *P. anea* were significant (*p* < 0.01). The trophic levels of *P. pawak* ranged from 3.70 to 4.21 based on the carbon and nitrogen stable isotope ratios, with an average of 3.97 and a trophic span of 0.51. The trophic levels of *P. anea* ranged from 3.30 to 4.28, with an average of 3.85 and a trophic span of 0.7. The trophic level of *P. pawak* showed a rising trend with the increase in the length, with a decreasing trend from the 131–150 mm length group. The trophic level of *P. anea* gradually increased with the body length (Figure 5). The trophic span of *P. pawak* is smaller than that of *P. anea*. With the increase in length group, the trophic level of *P. pawak* increased continuously, while that of *P. anea* decreased when it increased to a certain extent (Figure 5).

### 3.3. Trophic Niche

**Figure 6:**
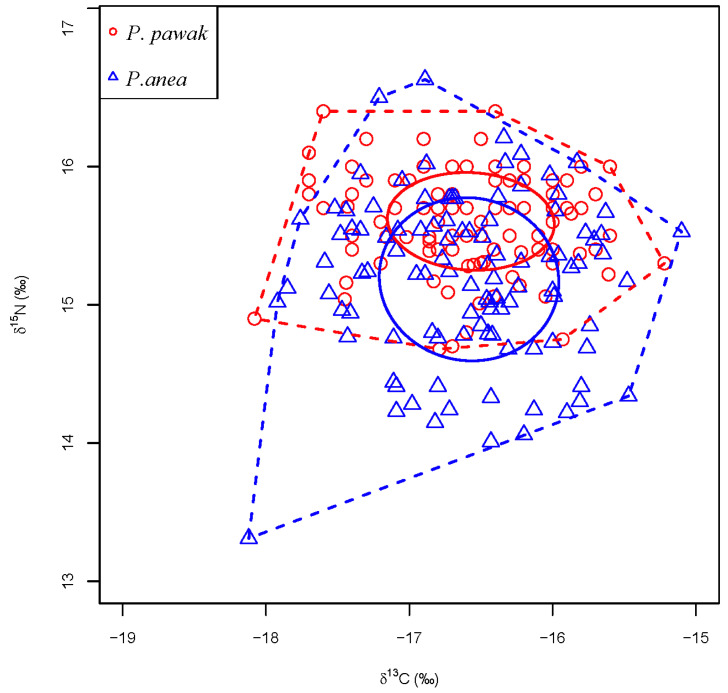
The trophic niches of two *Pennahia* species in the Beibu Gulf. The red circles represent *P. pawak*, and the blue triangles represent *P. anea*; the ellipses represent the core niche area, and the convex polygons represent the total niche area.

From the stable isotope distribution of carbon and nitrogen (Figure 6), it can be seen that the trophic niches of *P. pawak* and *P. anea* overlapped to some extent, but the degree of overlap was only 0.29, which was not significant. Based on the carbon and nitrogen stable isotope analysis, we calculated the trophic niche indexes of *P. pawak* and *P. anea*. The result showed that (Table 4) the basal food source, food chain length, level of trophic diversity, overall density, range of trophic niches, space of core niches, and total niche area of *P. anea* were larger than those of *P. pawak*.

### 3.4. Spatial Niche

**Figure 7:**
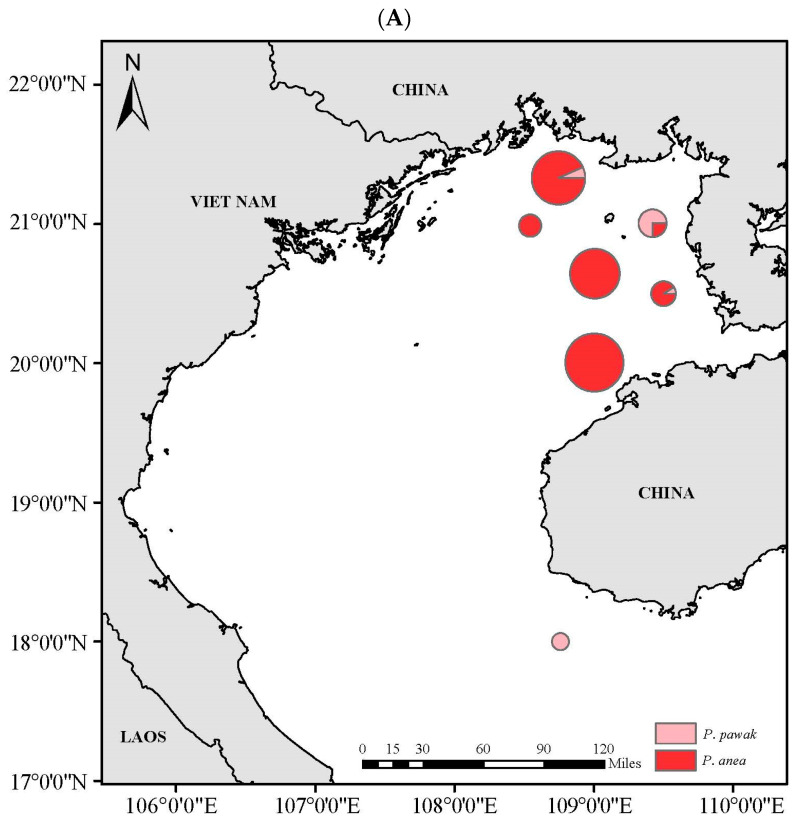

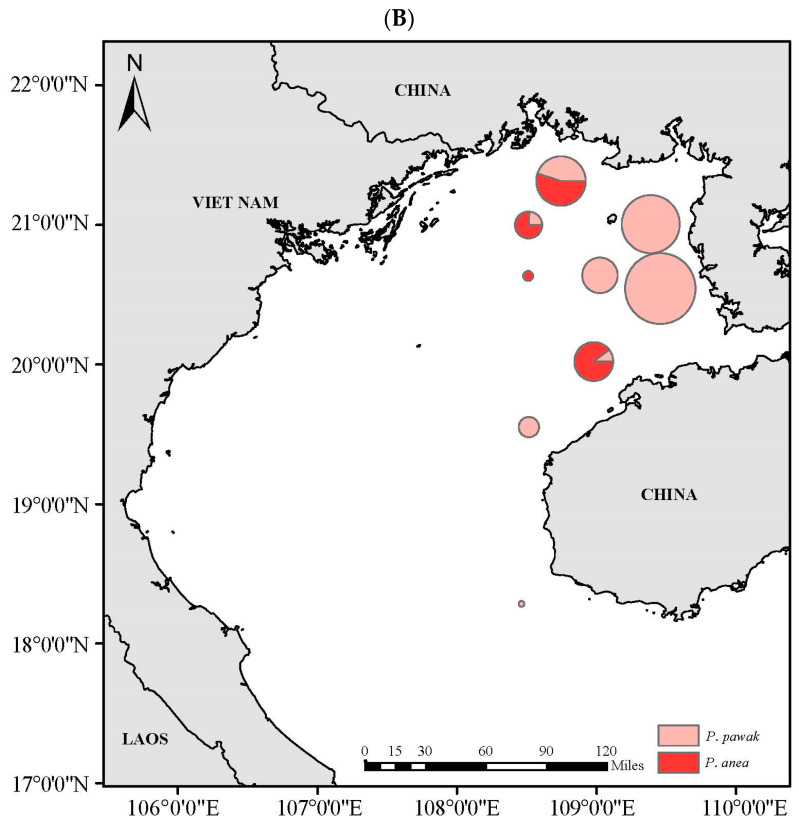
Resource density distribution of *Pennahia pawak (*Pink circle) and *Pennahia anea* (Red circle) in Beibu Gulf before (**A**) and after (**B**) SCS.

The resources of the Beibu Gulf of *P. pawak* and *P. anea* have obvious spatial distribution characteristics, and both of them are mainly distributed in the inner waters of the Beibu Gulf, while the *P. pawak* is also distributed in the middle and the mouth of the Gulf. *P. pawak* is distributed in the area of 108.46°~109.50° E, 18.00°~21.33° N, while *P. anea* is distributed in the area of 108.51°~109.50° E, 20.00°~21.33° N (Figure 7). The resource densities of the two species of *Pennahia* genus range from 0.08 to 109.52 kg/m^2^ and from 0.28 to 29.65 kg/m^2^, respectively, with the average resource density of *P. pawak* (21.19 kg/m^2^) being greater than that of *P. anea* (8.74 kg/m^2^). The spatial niche widths of *P. pawak* and *P. anea* differ before and after the summer close season, with *P. anea* having a greater niche width than *P. pawak* before the summer close season, while *P. pawak* had a greater spatial niche width than *P. anea* after the summer close season (Table 5). The spatial niche overlap index of the two *Pennahia* species was 0.20, which was present but not significant. Neither of the two *Pennahia* species reached a meaningful level of overlap before or after the summer close season(Table 5).

## 4. Discussion

This study revealed significant food overlap among individuals of different length groups in both *P. pawak* and *P. anea*. The optimal foraging theory suggests that individual feeding differences depend on the phenotype (size, sex, or developmental stage) and prey availability of the individual [41]. More precisely, although individuals may consume a wide variety of prey, they adopt more specialized feeding habits to avoid intraspecific competition, depending on the state of the individual and the bait resources available in the habitat. At low population densities, individuals compete for the same dominant prey, but as the population densities increase, this prey resource becomes scarce, and individuals begin to feed on other, different prey, suggesting that increasing population densities lead to increased intraspecific competition, which, in turn, increases the degree of feeding specialization [42]. Xia et al. [43] used a stomach content analysis and stable isotope analysis to study the feeding habits of *Megalobrama terminalis*, and the results showed that the decrease in intraspecific competition was mainly due to individual feeding specialization. However, in recent decades, the fishery resources in the Beibu Gulf have had a declining status due to over-exploitation by the fishery [44,45]. There is a significant overlap in the length group of the two species, indicating the potential presence of intraspecific competition. The degree of feeding specialization in both *P. pawak* and *P. anea* at the body length stage of 71–190 showed a decrease with the increase in the body length, indicating that there was a possible feeding shift at this stage.

The δ^13^C value is less variable during food chain transmission and can indicate its food source, while the δ^15^N value is relatively enriched in the organism and is generally used to determine the trophic level of the study object [35]. It has been shown that the carbon and nitrogen stable isotope compositions of fish are not constant at different growth stages [43]. In this study, the carbon stable isotope values of the two species did not vary much with the body length, which may be related to the small enrichment of carbon stable isotopes in the living body on the one hand [46], and on the other hand, although there was a shift in feeding, the main feeding taxa, especially the final trophic sources (elements), were relatively stable. In addition, the nitrogen stable isotopes and trophic levels showed overall increasing trends with the body length, which may be related to the increased feeding capacity of individuals. This pattern of change has been observed in many fish species [47,48], with the growth of individuals, the feeding and digestive organs of fish continuing to improve, the swimming ability continuing to increase, the range of prey selection becoming wider, and the prey of fish shifting from a low trophic level to a higher trophic level. Differences in prey resources across various sea regions can lead to variations in the trophic levels of fish. In a 2009 study on the predominant fish species in the Leizhou Bay area, Lu et al. observed that *P. pawak* exhibited a trophic level of 2.8 [49]. It is evident that the trophic level of *P. pawak* in Leizhou Bay is lower compared to that in the Beibu Gulf. Furthermore, this discrepancy may be associated with the size range of the collected samples. This study encompassed a diverse size range in the collected samples, potentially contributing to an overall higher trophic level for *P. pawak*.

The trophic niche width represents the strength of an organism’s utilization of habitat and resources, as well as its competitive ability [50,51]. In situations of limited resource supply, species with broader niches may exhibit stronger competitive abilities [52,53,54]. Strong interspecific competition causes species to reduce feeding on the same prey, thereby mitigating interactions with other species [55]. Niche overlap represents the frequency of encounters between two species on the same spatial resource, which can also reflect the potential competitive relationship between species [56]. In this study, the niche overlap indexes (*O_ij_* = 0.56 and *B_ij_* = 0.29) for both *P. pawak* and *P. anea* did not reach a significant level. Although niche overlap indicates potential interspecific competition, its intensity also depends on consumer and resource abundance [57]. In the present study, it was found that although Macrura organisms were the main prey for two *Pennahia* species, *P. anea* predominantly consumed *Alpheus*, while *P. pawak* consumed a percentage of *Metapenaeopsis barbata* and *Alpheus bisincisus* in addition to *Alpheus*. This suggests that *P. pawak* and *P. anea*, when they have the same prey base, show different feeding preferences by enhancing their feeding on different types of prey. This is consistent with the Evolutionarily Stable Strategy, in which closely related species inhabiting the same sea area will coexist by regulating their own feeding habits to attenuate food competition between species, thus maximizing equilibrium [13]. Based on the carbon and nitrogen stable isotope analysis, the difference in δ^13^C variation between *P. pawak* and *P. anea* was not significant, but there was no significant niche overlap between the two species, and the total niche width (TA) and the core niche (SEAc) of *P. anea* were larger than those of *P. pawak*. Combined with the analysis of food composition, although the food resources of the two species are generally similar, a differentiation in their trophic niches arises due to differences in their dominant feeding prey.

The width of the spatial niche reflects the spatial distribution of the species and its ability to utilize spatial resources [58]. The spatial niche width of *P. pawak* increased while that of *P. anea* decreased after the summer close season. *P. pawak* spawns in the April-August period [25], and the summer close season (May 1) facilitates the protection of the spawning population and juveniles, thus increasing its resource density. *P. anea* spawns in the March-June period [23], two months earlier than the summer close season. This is not enough to protect the spawning population and juveniles is insufficient, so its resource density is in a decreasing trend. Differences in the width of spatial niche is one of the important conditions for species to realize coexistence [58], and this change has a certain mitigating effect on the pressure of spatial resource competition between the two. According to the theory of resource competition [59], in an environment with limited resources, complete competitors cannot coexist, indicating niche differentiation along a certain resource dimension. The overlap between *P. pawak* and *P. anea* did not reach a significant level. The differences in their spatiotemporal distribution resulted in a low degree of overlap, reflecting a distinct niche differentiation between the two species.

The classical ecological theory suggests that the niche occupied by a species is limited by a variety of ecological factors, among which biological factors include its own population size, feeding and being fed on, and food competition, and that an increase in population size leads to increased competition [60]. When similar organisms with similar diets coexist in a resource-limited environment, dominant species eat whatever they want, while weaker competitors may be forced to eat different items. In marine ecosystems, marine organisms inhabiting the same sea area mainly reduce interspecific competition by realizing the differentiation of trophic, spatial, and temporal niches, among which the differentiation of trophic and spatial ecological niches is particularly important [57]. As demonstrated in the present study, in a resource-limited environment, coexisting species of *Pennahia* may potentially utilize the resources of the Beibu Gulf through the differentiation of their trophic and spatial ecological niches, and they may collectively utilize resources in the Beibu Gulf and achieve coexistence. In addition, the trophic and spatial niches of *P. pawak* are more advantageous than those of *P. anea*, suggesting a potentially stronger resource occupancy capability of *P. pawak*. This has important reference value for fishery management.

## 5. Conclusions

This study explores the feeding habits and ecological niches of *P. pawak* and *P. anea* in the Beibu Gulf. It highlights how individual feeding differences relate to prey availability, leading to specialized feeding habits to mitigate intraspecific competition. As population densities increase, competition intensifies, prompting individuals to shift their dominant prey. A stable isotope analysis revealed changes in trophic levels with the body length and differences in prey selection across growth stages. Despite having similarities in food resources, the two species exhibit distinct trophic niches due to differences in dominant prey. The spatial niche width also varies between the species, influenced by spawning seasons and resource availability. Niche differentiation aids in reducing interspecific competition, facilitating coexistence. These findings underscore the importance of trophic and spatial niche differentiation in marine ecosystems for species coexistence and have implications for fishery management.

## Figures and Tables

**Table 1:** Sampling information of two *Pennahia* species in Beibu Gulf in 2020.

Species	Number of Sample			
	Spring	Summer	Autumn	Winter
*P. pawak*	417	226	579	284
*P. anea*	317	241	788	388

**Table 2:** The diet overlap coefficient of *Pennahia pawak* and *Pennahia anea* in each length group. Values > 0.3 mean that the overlap is effective, and values > 0.6 represent significant overlap.

	*P. anea*	71–90	91–110	111–130	131–150	151–170	171–190
*P. pawak*							
71–90			0.82	0.66	0.68	0.61	0.93
91–110		0.99		0.88	0.83	0.77	0.63
111–130		0.99	0.99		0.84	0.87	0.38
131–150		0.83	0.85	0.88		0.91	0.55
151–170		0.78	0.80	0.84	0.98		0.40
171–190		0.59	0.61	0.65	0.89	0.90	

**Table 3:** The diet compositions of the *Pennahia pawak* and *Pennahia anea* captured in the Beibu Gulf. The F% (frequency of occurrence), W% (percentage in biomass), N% (percentage of number), IRI (index of relative importance), and IRI% (index of relative importance expressed as a percentage) values are shown to provide insight into each prey item. “+” indicates that the ratio was smaller than 0.01%, and “-” indicates absence.

Prey	*P. pawak*				*P. anea*			
Science Name	W%	N%	F%	IRI%	W%	N%	F%	IRI%
Pisces								
*Leiognathidae*	0.46	0.22	0.56	0.02	0.42	0.92	3.86	0.03
*Trichiuridae*	0.87	0.88	2.22	0.18	0.47	0.23	0.97	+
*Thrissa dussumieri*	2.90	0.44	1.11	0.17	3.02	0.69	2.90	0.07
*Champsodon atridorsalis*	0.35	0.66	1.67	0.08	0.10	0.12	0.48	+
*Photopectoralis bindus*	1.98	2.84	7.22	1.60	1.09	0.69	2.90	0.03
*Stolephorus heterolobas*	0.66	0.44	1.11	0.06	0.23	0.12	0.48	+
*Secutor ruconius*	0.23	0.22	0.56	0.01	0.83	0.35	1.45	0.01
*Bregmaceros rarisquamosus*	1.54	6.35	16.11	5.82	8.07	23.44	98.07	19.14
*Apogonidae*	4.52	6.13	15.56	7.60	0.21	0.12	0.48	+
*Bregmaceros*	3.25	7.88	20.00	10.20	9.07	28.52	119.32	27.79
*Gobiidae*	2.44	3.50	8.89	2.42	0.05	0.12	0.48	+
*Atherinidae*	0.33	0.22	0.56	0.01	1.55	0.46	1.93	0.02
*Bregmaceros nectabanus*	3.08	3.94	10.00	3.22	6.20	7.51	31.40	2.67
*Stolephorus indicus*	0.23	0.22	0.56	0.01	1.26	0.35	1.45	0.01
*Stolephorus*	2.75	2.41	6.11	1.45	47.16	26.10	109.18	49.55
*Champsodon*	0.63	0.88	2.22	0.15	-	-	-	-
*Jaydia striata*	0.11	0.22	0.56	0.01	-	-	-	-
*Trypauchen vagina*	0.94	0.66	1.67	0.12	-	-	-	-
*Thryssa*	0.21	0.22	0.56	0.01	-	-	-	-
*Bregmaceros macclellandii*	0.59	0.22	0.56	0.02	-	-	-	-
*Diaphus knappi*	0.11	0.22	0.56	0.01	-	-	-	-
*Parachaeturichthys polynema*	2.56	1.53	3.89	0.73	-	-	-	-
*Carangidae*	0.54	0.22	0.56	0.02	-	-	-	-
*Leiognathus berbis*	0.67	0.22	0.56	0.02	-	-	-	-
*Sirembo imberbis*	0.22	0.22	0.56	0.01	-	-	-	-
*Callionymus octostigmatus*	1.15	0.22	0.56	0.03	-	-	-	-
*Sirembo*	0.04	0.22	0.56	0.01	-	-	-	-
*Hypoatherina valenciennei*	-	-	-	-	0.64	0.12	0.48	+
*Leiognathus nuchalis*	-	-	-	-	0.08	0.12	0.48	+
*Stolephorus commersonnii*	-	-	-	-	4.59	1.62	6.76	0.26
*Stolephorus zollingeri*	-	-	-	-	0.60	0.23	0.97	+
*Sardinella*	-	-	-	-	6.18	0.92	3.86	0.17
*Stolephorus chinensis*	-	-	-	-	0.56	0.35	1.45	0.01
Macrura								
*Alpheus*	5.16	16.19	41.11	40.26	1.05	1.50	6.28	0.10
*Metapenaeopsis palmensis*	0.85	1.09	2.78	0.25	0.48	0.23	0.97	+
*Alpheus bisincisus*	3.57	5.47	13.89	5.76	0.70	0.35	1.45	0.01
*Metapenaeopsis barbata*	9.47	7.00	17.78	13.43	0.52	0.12	0.48	-
*Solenocera crassicornis*	2.55	1.75	4.44	0.88	0.46	0.12	0.48	-
*Metapenaeopsis*	0.70	1.31	3.33	0.31	-	-	-	-
*Alpheus brevicristatus*	0.84	1.31	3.33	0.33	-	-	-	-
*Penaeidae*	1.59	1.31	3.33	0.44	-	-	-	-
*Parapenaeopsis*	0.10	0.44	1.11	0.03	-	-	-	-
*Parapenaeopsis*	0.51	0.22	0.56	0.02	-	-	-	-
*Metapenaeopsis acclivis*	0.56	0.22	0.56	0.02	-	-	-	-
*Parapenaeus sextuberculatus*	0.43	0.44	1.11	0.04	-	-	-	-
*Parapenaeus*	0.17	0.22	0.56	0.01	-	-	-	-
*Alpheus japonicus*	0.22	0.44	1.11	0.03	-	-	-	-
*Alpheus distinguendus*	1.21	1.75	4.44	0.60	-	-	-	-
*Metapenaeus*	0.06	0.22	0.56	0.01	-	-	-	-
*Trachypenaeus curvirostris*	0.26	0.44	1.11	0.04	-	-	-	-
*Palaemonidae*	0.03	0.22	0.56	0.01	-	-	-	-
*Miyadiella podophthalmus*	0.02	0.44	1.11	0.02	-	-	-	-
*Parapenaeus longipes*	0.68	0.88	2.22	0.16	-	-	-	
*Parapenaeopsis cornuta*	-	-	-	-	0.54	0.46	1.93	0.01
*Trachypenaeus pescadoreensis*	-	-	-	-	0.52	0.12	0.48	+
*Parapenaeopsis incisa*	-	-	-	-	0.21	0.23	0.97	+
Brachyura								
*Portunus*	0.18	1.31	3.33	0.23	0.03	0.12	0.48	-
*Eucrate alcocki*	0.06	0.22	0.56	0.01	-	-	-	-
*Typhlocarcinops canaliculata*	0.05	0.22	0.56	0.01	-	-	-	-
*Charybdis hellerii*	0.21	0.44	1.11	0.03	-	-	-	-
*Charybdis variegata brevispinosa*	0.55	0.66	1.67	0.09	-	-	-	-
*Lissocarcinus laevis*	0.03	0.22	0.56	0.01	-	-	-	-
*Portunus hastatoides*	0.58	2.19	5.56	0.70	-	-	-	-
*Jonas*	0.04	0.22	0.56	0.01	-	-	-	-
*Eucrate solaris*	0.12	0.22	0.56	0.01	-	-	-	-
*Charybdis*	0.25	0.88	2.22	0.11	-	-	-	-
*Petrolisthes*	0.05	0.22	0.56	0.01	-	-	-	-
*Portunus argentatus*	1.43	2.41	6.11	1.07	-	-	-	-
*Charybdis truncata*	0.07	0.22	0.56	0.01	-	-	-	-
*Carcinoplax purpurea*	0.10	0.22	0.56	0.01	-	-	-	-
*Charybdis vadorum*	0.06	0.22	0.56	0.01	-	-	-	-
*Charybdis variegata*	0.10	0.44	1.11	0.03	-	-	-	-
*Typhlocarcinus villosusStimpson*	-	-	-	-	0.03	0.12	0.48	-
Stomatopoda								
*Gryllotalpidae*	0.16	0.22	0.56	0.01	0.09	0.12	0.48	+
*Oratosquillina interrupta*	0.48	0.44	1.11	0.05	-	-	-	-
Oratosquilla oratoria	0.35	0.44	1.11	0.04	-	-	-	-
*Anchisquilla fasciata*	0.16	0.22	0.56	0.01	-	-	-	-
*Oratosquillina*	0.28	0.44	1.11	0.04	-	-	-	-
*kempina stridulans*	0.77	0.66	1.67	0.11	-	-	-	-
*Squillidae*	-	-	-	-	0.23	0.92	3.86	0.03
Cephalopoda								
*Cephalopoda*	0.17	1.53	3.89	0.30	2.00	0.69	2.90	0.05
Isopoda								
*isopoda*	0.15	0.88	2.22	0.10	0.04	0.23	0.97	+
Gastropoda								
*Turritella terebra*	0.32	1.53	3.89	0.33	-	-	-	-
Polychaeta								
*Nereis*	0.14	0.22	0.56	0.01	-	-	-	-
*Sipunculs nudus*	0.23	0.44	1.11	0.03	-	-	-	-

**Table 4:** The trophic niche indicators of two *Pennahia* species in the Beibu Gulf. The CD (centrifugal distance), CR (carbon range), NR (nitrogen range), MNND (mean nearest neighbor distance), SDNND (standard deviation of nearest neighbor distance), SEAc (standard ellipse corrected area), TA (the area of the convex hull)values are shown to provide insight into the trophic niches. An overlap index of >0.3 means that the overlap is effective, and an overlap index of >0.6 represents significant overlap.

Species	CD	CR	NR	MNND	SDNND	SEAc	TA	Overlap Index
*P. pawak*	0.59	2.86	1.72	0.09	0.09	0.65	3.70	0.29
*P. anea*	0.76	3.02	3.32	0.13	0.15	1.16	6.09	

**Table 5:** Spatial niche width and overlap index of two *Pennahia* species in Beibu Gulf; SCS indicates summer close season. Overlap index of >0.3 means overlap is effective, and overlap index of >0.6 represents significant overlap.

		Total	Before SCS	After SCS
Spatial niche width	*P. pawak*	1.19	0.10	1.22
	*P. anea*	0.58	0.85	0.50
Overlap index		0.20	0.16	0.13

## Data Availability

The data presented in this study are available on request Konglan Luo (email: luokonlan@gmail.com). The data are not publicly available due to [the need to reserve the data for future research use].

## References

[1] Liu J. (1999). Advanced Aquatic Biology.

[2] Brönmark C., Hansson L. (2005). The Biology of Lakes and Ponds.

[3] Ward A., Webster M., Hart P. (2006). Intraspecific Food Competition in Fishes. Fish Fish..

[4] Deng J., Jiang W., Yang J., Li J. (1997). Species Interaction and Food Web of Major Predatory Species in the Bohai Sea. J. Fish. Sci. China.

[5] Tang Q., Fan Y., Lin H. (1996). Initial Inquiring Into the Developmental Strategy of Chinese Ocean Ecosystem Dynamics Research. Adv. Earth Sci..

[6] Wang J., Su Y., Liu J., Qiu X. (1994). The Feeding Habits of Five Sciaenids Fishes in Louyuan Bay. J. Jimei Univ. (Nat. Sci.).

[7] Li Z., Dai Q., Zuo T., Jin X., Zhuang Z. (2009). Studies On Food Competition between *Pseudosciaena polyactis* and *Johnius belengerii* From Changjian Estuary and Adjacent Southern Yellow Sea in Autumn. J. Hydroecol..

[8] Matley J., Heupel M., Fisk A., Simpfendorfer C., Tobin A. (2017). Measuring Niche Overlap Between Co-Occurring *Plectropomus* spp. Using Acoustic Telemetry and Stable Isotopes. Mar. Freshw. Res..

[9] Wang Q., Liu M., Zhu F., Li Z., Liu S., Duan X., Chen D., Yang R. (2019). Comparative Study of Three Species of Schizothoracine On Feeding and Digestive Organs in Upper Nujiang River. Chin. J. Zool..

[10] Zhang Y., Xu B., Zhang C., Ji Y., Cheng Y., Xue Y. (2020). Spatial Heterogeneity in the Feeding Habits and Feeding Ground Distribution of *Johnius belangerii* in Haizhou Bay During Spring. J. Fish. Sci. China.

[11] Olesen J.M., Bascompte J., Dupont Y.L., Jordano A.P. (2007). The Modularity of Pollination Networks. Proc. Natl. Acad. Sci. USA.

[12] Raul C.P., MaRcio S.A., Souza F.L., Ingram T. (2019). Competition and Resource Breadth Shape Niche Variation and Overlap in Multiple Trophic Dimensions. Proc. Biol. Sci..

[13] Yunkai L., Huiqiong W., Xinjun C., Yi G. (2020). Trophic Niches and Interspecific Relationships Between Closely Related Ommastrephidae Species, *Dosidicus gigas* and *Sthenoteuthis oualaniensis*. Acta Ecol. Sin..

[14] Xiao Y., Jiang R., Yin R., Wang J., Yang F., Wang H., Li Z. (2023). Trophic Niche and Interspecific Relationship of Five Eels in the Waters of the Zhoushan Islands. J. Fish. China.

[15] Yang Z., Chen X., Zhao N., Tang H., Tao J., Zhang P., Fang S., Wan C. (2018). The Effect of Different Habitat Types and Ontogenetic Stages On the Diet Shift of a Critically Endangered Fish Species, *Coreius guichenoti* (Sauvage and Dabry De Thiersant, 1874). Int. J. Environ. Res. Public Health.

[16] Hyslop E.J. (1980). Stomach Contents Analysis—A Review of Methods and their Application. J. Fish Biol..

[17] Xue Y., Jin X. (2003). Review of the Study On Feeding Habits of Fishes and Food Webs. Prog. Fish. Sci..

[18] Dixon H., Dempson J., Sheehan T., Renkawitz M., Power M. (2017). Assessing the Diet of North American Atlantic Salmon (*Salmo salar L.*) Off the West Greenland Coast Using Gut Content and Stable Isotope Analyses. Fish Ocean..

[19] Wang X., Qiu Y., Du F., Lin Z., Sun D., Huang S. (2012). Dynamics of Demersal Fish Species Diversity and Biomass of Dominant Species in Autumn in the Beibu Gulf, Northwestern South China Sea. Acta Ecol. Sin..

[20] Yi X., Qiu K., Zhou X., Zhao C., Deng Y., He X., Yan Y. (2021). Analysis of Fishery Biology of *Pennahia pawak* in Beibu Gulf. J. Shanghai Ocean Univ..

[21] Wang X., Du F., Qiu Y. (2006). Length-Weight Relationships of Important Commercial Fishes in Northern South China Sea. J. Appl. Oceanogr..

[22] Wagiyo K., Tirtadanu, Chodriyah U. (2020). Biology Characteristic, Abundance Index and Fishing Aspect of Donkey Croaker (*Pennahia anea* Bloch, 1793) in the Tangerang Waters. E3S Web Conf..

[23] Tuuli C., de Mitcheson Y., Liu M. (2011). Reproductive Biology of the Greyfin Croaker *Pennahia anea* in the Northern South China Sea. Ichthyol. Res..

[24] He X., Tao Y., Hou G., Lu H., Yan Y. (2015). Population Structure and Spatio-Temporal Distribution of *Pennahia pawak* in the Beibu Gulf, South China Sea. J. Guangdong Ocean Univ..

[25] Yan Y., Hou G., Lu H., Yin Q. (2011). Age and Growth of Pawak Croaker *Pennahia pawak* in Beibu Gulf. J. Fish. Sci. China.

[26] Yi X. (2021). Feeding Ecology of Pawak Croaker (*Pennahia pawak*) in the Beibu Gulf. Master’s Thesis.

[27] Third Institute of Oceanography State Oceanic Administration (2007). Specifications for Oceanographic Survey—Part 6: Marine Biological Survey. General Administration of Quality Supervision, Inspection and Quarantine of the People’s Republic of China.

[28] Figueiredo M., Morato T., Barreiros J.P., Afonso P., Santos R.S. (2005). Feeding Ecology of the White Seabream, *Diplodus sargus*, and the Ballan Wrasse, *Labrus bergylta*, in the Azores. Fish. Res..

[29] Dou S. (1992). Fish-Stomach Content Analysis: Methods and Application. Mar. Sci. Bull..

[30] Pianka E. (1973). The Structure of Lizard Communities. Annu. Rev. Ecol. Evol. Syst..

[31] Shannon C. (1997). The Mathematical Theory of Communication. MD Comput..

[32] Roughgarden J. (1972). Evolution of Niche Width. Am. Nat..

[33] Bolnick D.I., Yang L.H., Fordyce J.A., Davis J.M., Svanbäck R. (2002). Measuring Individual-Level Resource Specialization. Ecology.

[34] McKinney C., McCrea J., Epstein S., Allen H., Urey H. (1950). Improvements in Mass Spectrometers for the Measurement of Small Differences in Isotope Abundance Ratios. Rev. Sci. Instrum..

[35] Post D. (2002). Using Stable Isotopes to Estimate Trophic Position: Models, Methods, and Assumptions. Ecology.

[36] He X., Zhu D., Zhao C., Yan Y., Kang B. (2019). Feeding Habit of Asian Moon Scallop (*Amusium pleuronectes*) and as an Isotopic Baseline Indicator in the Beibu Gulf, South China Sea. J. Shellfish Res..

[37] Jackson A., Inger R., Parnell A., Bearhop S. (2011). Comparing Isotopic Niche Widths among and within Communities: Siber-Stable Isotope Bayesian Ellipses in R. J. Anim. Ecol..

[38] Schoener T.W. (1968). Sizes of Feeding Territories Among Birds. Ecology.

[39] Zhan B. (1995). Fishery Resources Assessment.

[40] Jensen H., Kiljunen M., Amundsen P.A. (2012). Dietary Ontogeny and Niche Shift to Piscivory in Lacustrine Brown Trout Salmo Trutta Revealed by Stomach Content and Stable Isotope Analyses. J. Fish Biol..

[41] Schoener T. (1974). Resource Partitioning in Ecological Communities: Research On How Similar Species Divide Resources Helpsreveal the Natural Regulation of Species Diversity. Science.

[42] Svanbäck R., Bolnick D.I. (2005). Intraspecific Competition Affects the Strength of Individual Specialization: An Optimal Diet Theory Method. Evol. Ecol. Res..

[43] Xia Y., Li Y., Zhu S., Li J., Li S., Li X. (2020). Individual Dietary Specialization Reduces Intraspecific Competition, Rather than Feeding Activity, in Black Amur Bream (*Megalobrama terminalis*). Sci. Rep..

[44] Sun D. (2008). A Study On Fishery Resources and Sustainable Fishery Development in the Beibu Bay. Master’s Thesis.

[45] Su L., Chen Z., Zhang K., Xu Y., Qiu Y. (2021). Establishment of Quality Status Evaluation System of Fishery Resourcesin Beibu Gulf Based On Bottom Trawl Survey Data. J. Guangdong Ocean Univ..

[46] Zhang B., Yuan W., Dai F. (2016). Study On Feeding Ecology of Fish Community in Laoshan Bay During Summer Using Stable Carbon and Nitrogen Isotopes. J. Fish. China.

[47] Ji W., Chen X., Jiang Y., Liu Y., Hu F., Li S. (2011). Stable Isotope Analysis of some Representative Nektonic Organisms in the Central and Northern Part of East China Sea. Mar. Fish..

[48] Gao Y., Sui H., Ren X., Xu B., Zhang C., Ren Y., Xue Y. (2020). Feeding Habits of *Saurida elongata* in Haizhou Bay, Shandong, China, Based On Stomach Contents and Stable Isotope. Chin. J. Appl. Ecol..

[49] Lu H., Ou F., Yan Y., Zhang J. (2009). Study On Trophie Level of Main Fishes in the Leizhou Baywith Stable Nitrogen Isotope Techniques. Acta Ocean. Sin..

[50] Li Y., Gao X., Wang L., Fang L. (2018). Trophic Niche Partitioning of Pelagic Sharks in Central Eastern Pacific Inferred From Stable Isotope Analysis. Chin. J. Appl. Ecol..

[51] Li Y., Chen Z., Gong Y., Chen X. (2021). A Review On the Methods Used in Trophic Niche Studies of Marine Animals and their Applications. J. Trop. Oceanogr..

[52] SVANBÄCK R., PERSSON L. (2004). Individual Diet Specialization, Niche Width and Population Dynamics: Implications for Trophic Polymorphisms. J. Anim. Ecol..

[53] Li L., Jin X., Ma B., Wu S., Tang S. (2020). Trophie Niche and Interspecifie Diet Relationship of *Gymnocypris* in Autumn from Langcuo Lake of Tibet, China. Chin. J. Appl. Ecol..

[54] Ge S., Zhao W., Song J., Yu D., Liu Y., Wang Q., Zhou J. (2019). Study On Trophic Niches of *Sebastes schlegelii* and *Hexagrammos otakii* in the Artificial Reef Area of Xiaoheishan Island. Acta Ecol. Sin..

[55] Abrams P. (1980). Some Comments On Measuring Niche Overlap. Ecology.

[56] Yang X., Ma J. (1992). A Review On some Terms Related to Niche and their Measurements. Chin. J. Ecol..

[57] Hurlbert S.H. (1978). The Measurement of Niche Overlap and some Relatives. Ecology.

[58] Yu Z., Jin X., Li X. (2010). Analysis of Ecological Niche for Major Fish Species in the Central and Southern Yellow Sea. Prog. Fish. Sci..

[59] Nan C., Dong S. (2003). A Review On Resource Competition Theory. Chin. J. Ecol..

[60] Svanback R., Bolnick D.I. (2007). Intraspecific Competition Drives Increased Resource Use Diversity within a Natural Population. Proc. R. Soc. B Biol. Sci..

